# Magnetic resonance microscopy of renal and biliary abnormalities in excised tissues from a mouse model of autosomal recessive polycystic kidney disease

**DOI:** 10.14814/phy2.12517

**Published:** 2015-08-28

**Authors:** Choong H Lee, Amber K O’Connor, Chaozhe Yang, Joshua M Tate, Trenton R Schoeb, Jeremy J Flint, Stephen J Blackband, Lisa M Guay-Woodford

**Affiliations:** 1 Department of Neuroscience, University of FloridaGainesville, Florida; 2 McKnight Brain Institute, University of FloridaGainesville, Florida; 3 Center for Translational Science, Children’s National Health SystemWashington, District of Columbia; 4 Department of Genetics, University of Alabama at BirminghamBirmingham, Alabama; 5 National High Magnetic Field LaboratoryTallahassee, Florida

**Keywords:** Autosomal recessive polycystic kidney disease, *cpk* mouse, magnetic resonance microscopy, polycystic kidney disease

## Abstract

Polycystic kidney disease (PKD) is transmitted as either an autosomal dominant or recessive trait and is a major cause of renal failure and liver fibrosis. The *cpk* mouse model of autosomal recessive PKD (ARPKD) has been extensively characterized using standard histopathological techniques after euthanasia. In the current study, we sought to validate magnetic resonance microscopy (MRM) as a robust tool for assessing the ARPKD phenotype. We used MRM to evaluate the liver and kidney of wild-type and *cpk* animals at resolutions <100 *μ*m and generated three-dimensional (3D) renderings for pathological evaluation. Our study demonstrates that MRM is an excellent method for evaluating the complex, 3D structural defects in this ARPKD mouse model. We found that MRM was equivalent to water displacement in assessing kidney volume. Additionally, using MRM we demonstrated for the first time that the *cpk* liver exhibits less extensive ductal arborization, that it was reduced in volume, and that the ductal volume was disproportionately smaller. Histopathology indicates that this is a consequence of bile duct malformation. With its reduced processing time, volumetric information, and 3D capabilities, MRM will be a useful tool for future in vivo and longitudinal studies of disease progression in ARPKD. In addition, MRM will provide a unique tool to determine whether the human disease shares the newly appreciated features of the murine biliary phenotype.

## Introduction

Polycystic kidney disease (PKD) is characterized by the development of fluid-filled cysts in the kidneys and liver. PKD affects more than 600,000 people in the United States and is a major cause of end-stage renal disease (Torres and Harris 2012). The two types of PKD, autosomal dominant (MIM 173900) and autosomal recessive (MIM 263200), are distinguished by their mode of inheritance, disease-associated genes, and pathologic features. Autosomal recessive PKD (ARPKD) results from mutations in the *PKHD1* gene (Torres and Harris 2007) and is relatively infrequent, occurring in 1:20,000 live births (Zerres et al. 1998). ARPKD is a severe, early-onset disease that primarily involves the kidneys and biliary tract (Guay-Woodford 2003). The majority of patients are identified in utero or as infants with enlarged and echogenic kidneys. The main pathological features of ARPKD are the dilation of the renal collecting ducts, biliary dysgenesis, and portal tract fibrosis. Postnatal morbidity results from severe systemic hypertension, renal insufficiency, and portal hypertension due to portal-tract dysgenesis and associated fibrosis. In terms of the liver phenotype, the data from histopathological and sonographic studies reveal features of biliary tract fibrosis (sometimes with associated Caroli’s disease). However, the three-dimensional (3D) intricacies of the ARPKD-associated biliary defects have not been previously defined in high resolution.

When first detected in utero, ARPKD kidneys are hyperechogenic on sonography and display decreased cortico-medullary differentiation due to collecting duct dilatation (Chaumoitre et al. 2006). However, beyond 2–4 years of age, renal size decreases in ARPKD patients due to the combined effects of renal fibrosis and nephron loss (Blickman et al. 1995). In contrast, disease progression in ADPKD is typically associated with progressively increasing kidney size. The Consortium for Radiologic Imaging Studies of Polycystic Kidney Disease (CRISP) has demonstrated that MRI-based volumetric measurements are predictive with respect to the severity of ADPKD progression (Chapman et al. 2003). However, to date in ARPKD, the highly variable morphology of the renal and biliary lesions has confounded efforts to use imaging modalities to monitor disease progression as either predictive or prognostic markers of disease progression (Chaumoitre et al. 2006; Turkbey et al. 2009). Moreover, while MRI imaging allows for high reproducibility and accuracy in quantitative analysis without exposure to ionizing radiation (Laing and Gibson 1998), in infants and young children who cannot remain still while being imaged, traditionally the utility of MRI is offset by the need for sedation. Latex-based CT has been used to evaluate the architecture of the biliary phenotype in rats (Masyuk et al. 2004), however, with its superior contrast in soft tissues and recent technological advances, magnetic resonance imaging shows greater promise. Indeed, we and others expect that over the next several years, MRI will become the imaging modality of choice for patients with structural diseases of the kidney and liver.

A number of animal models, primarily mice and rats, have been used to characterize the recessive PKD phenotype. To date, models developed through targeted disruption of *Pkhd1*, the mouse ortholog of the human ARPKD gene, present with limited to no renal phenotype. In contrast, the *cpk* mouse model that results from a mutation in the nonorthologous gene *Cys1*, closely phenocopies human ARPKD in terms of both the renal and the biliary lesion. It remains the most extensively characterized ARPKD mouse model to date (Preminger et al. 1982; Fry et al. 1985; Guay-Woodford 2003). One key distinction between the human ARPKD patients and the *cpk* model is that the disease in the mouse is more rapidly progressive with mutant mice dying by 21 days of age with severely enlarged kidneys and renal insufficiency (Gattone et al. 1988). Water displacement studies indicate that a *cpk* kidney is roughly eight times larger than an age-matched healthy kidney (Mrug et al. 2005).

In this study, we examined the utility of high-resolution MRI to characterize the cystic structures in excised kidneys and livers of *cpk* and control mice. The small size of these excised samples facilitated the use of small detector coils so that higher spatial resolutions could be achieved (<100 *μ*m). At these resolutions, MRI is referred to as magnetic resonance microscopy (MRM) (Tyszka et al. 2005). The soft-tissue contrast and the microscopic resolution in both organs yielded reproducible, quantitative data that we converted into 3D renderings. In addition, we calculated the volume of the *cpk* kidneys from the MRM data and compared the accuracy of the MRM-derived volumes to measurements obtained via water displacement, finding that the two methods were strongly correlated.

In assessing the intact, 3D biliary tree with its ductal structures and associated portal vein ramifications, we also observed several novel aspects of the *cpk* biliary phenotype. Previous two-dimensional histopathological characterization of the recessive PKD liver demonstrated that the associated ductal plate malformation results in chaotic, widely dilated biliary ducts (Wen 2011; Luoto et al. 2014). The new findings from our MRM-based studies indicate that despite this ductal dilation, the intrahepatic biliary arborization is blunted and at the age that we collected tissues, before the onset of secondary fibrosis, the *cpk* liver is actually smaller than its wild-type counterpart. Together, these data provide new insights into the pathogenesis of recessive PKD and in particular, a new starting point to understand the development and progression of the biliary disease.

## Materials and Methods

### Mice and histological processing

The mice used in these experiments were maintained at the University of Alabama at Birmingham (UAB), and were bred from BALB/c:C57BL6/J *Cys1*^*cpk*^ mice (in which the *Cys1*^*cpk*^ allele was introgressed onto the BALB/c background). Homozygous mutants express both severe renal and biliary disease (Gattone et al. 2002). F1 progeny heterozygous for the *Cys1*^*cpk*^ allele were identified using standard PCR-based genotyping techniques and intercrossed to generate F2 controls (*+/+* or *cpk/+*) and mutant mice (*cpk/cpk*). Mice were euthanized at 20–22 days of age. Both kidneys and liver were fixed in 10% buffered formalin for 2 days prior to analysis.

After water displacement and MRM analyses (see below), the tissues were embedded in paraffin and five micron sections were stained with hematoxylin and eosin (H&E). Images of portal areas were captured with a SPOT Insight digital camera (SPOT Imaging Solutions, Sterling Heights, MI) and bile duct number, bile duct area, portal vein area, and connective tissue area were determined using Image Pro Plus v6 software (Media Cybernetics, Inc. Rockville, MD) (Table3; Fig.4). We developed a standardized protocol for processing the livers using anatomic landmarks to ensure that the sections from wild-type and *cpk* livers corresponded as closely as possible to the same regions of the median lobe. All protocols were approved by the UAB Animal Care and Use Committee. UAB is fully accredited by the American Association of the Accreditation of Laboratory Animal Care.

### Water displacement

Kidney volume (*cpk* mice) was first measured by water displacement ex vivo before MRM, as described previously (Mrug et al. 2005). Volume measurements were taken after the kidneys had been fixed and then washed/equilibrated in phosphate-buffered saline (PBS). Due to the small size of the wild-type kidneys, reproducible measurements of their volume could not be obtained using the water displacement method (Table1).

**Table 1:** Kidney volumes as determined by MRM and water displacement

(A) Wild-type kidney			
Sample no.	Pixel count	MRM Vol (mm^3^)	Displacement (mm^3^)
1	23,278	102.3	nd
2	18,292	80.38	nd
3	22,771	100.07	nd
4	19,313	84.87	nd
5	23,237	102.12	nd
6	22,597	99.3	nd
7	21,932	96.38	nd
8	17,855	78.46	nd
Mean		92.99	
SD		10.05	
SEM		3.55	

(B) Mutant kidney			
Sample no.	Pixel count	MRM Vol (mm^3^)†	Displacement (mm^3^)†
1	131,991	1611.22	1550
2	104,608	1276.95	1250
3	84,629	1033.07	1050
4	111,365	1359.44	1350
5	97,025	1184.39	1150
6	110,130	1344.36	1350
7	79,227	967.13	950
8	118,123	1441.93	1350
9	117,089	1429.31	1350
10	109,599	1337.88	1450
11	119,089	1453.72	1450
12	102,556	1252.9	1250
Mean		1307.61	1291.67
SD		181.37	172.99
SEM		52.36	49.94

MRM, magnetic resonance microscopy; nd, not determined; SD, standard deviation; SEM, standard error of the mean.

† Bland–Altman analysis indicates that the MRM values are as good as the displacement values (see Materials and Methods section for details).

### Kidney and liver MRM

After water displacement measurements, MRM was performed with a Bruker 14.1 Tesla (T) and 17.6 T vertical magnets (for the *cpk* kidney only) both equipped with 3000 mT/m gradients and interfaced with a 10-mm bird cage coil. Two wild-type kidneys, wrapped in a Kimwipe (Kimberly-Clark, Roswell, GA) to prevent movement during scanning, were easily accommodated in the 10-mm diameter sample tubes. However, due to the enlarged features of the diseased kidneys and the resulting fragility of the tissue, extra care was taken when positioning the mutant samples into the sample tubes. Scanning of the diseased kidneys was conducted in a 17.6 T magnet. For liver imaging, the median lobe, the largest of the four lobes that comprise the mouse liver, of both the wild-type and *cpk* livers were imaged with the 14.1 T magnet. Images were acquired at ambient temperature (22.0 ± 0.5°C).

For volume measurements of the kidneys and livers, 2D multislice multiecho (MSME) MRM was employed. For the kidney imaging, 2D MSME MRM both in coronal and sagittal view (echo time [TE] = 100 msec, repetition time [TR] = 2000 msec, number of averages = 2, field of view [FOV] = 20 × 20 mm, sampling matrix = 256 × 256, slice thickness = 200 *μ*m, in-plane resolution = 78 *μ*m) were acquired. For the liver imaging, 2D MSME images both in coronal and sagittal view (TE = 50 msec, TR = 2500 msec, number of averages = 10, FOV = 20 × 10 mm, sampling matrix = 256 × 128, slice thickness = 200 *μ*m, in-plane resolution = 78 *μ*m) were collected. Total kidney volume was determined using segmentation analysis (AMIRA 3.1.1; Mercury Computer Systems Reston, VA). Tissues were manually outlined and segmented in AMIRA. The number of pixels from segmented tissue within each slice were added together to produce a total pixel count (TPC) for each tissue type. The total volume (TV) was then calculated by the following formula: TV = TPC × (in-plane resolution) × (slice thickness). In the livers, the biliary tree volume was calculated automatically by the software after manual definition of the duct boundaries (Table2; Fig.3).

**Table 2:** Ductal volumes as determined by MRM

Sample	DV (mm^3^)	TV (mm^3^)	DV/TV ratio
(A) Wild-type liver (median lobe)			
1	124.79	648.19	0.1925
2	119.13	605.21	0.1968
3	118.78	613.41	0.1936
4	108.91	770.68	0.1413
5	100.87	784.49	0.1286
6	98.01	565.03	0.1734
Mean	111.75	664.51	0.1710*
SD	10.85	91.61	0.0294
SEM	4.43	37.40	0.0120
(B) Mutant liver (median lobe)			
1	10.32	115.80	0.0892
2	59.62	411.34	0.1450
3	29.43	364.10	0.0808
4	40.20	377.11	0.1066
5	35.68	384.04	0.0929
6	93.36	594.38	0.1571
Mean	44.77	374.46	0.1119*
SD	28.66	152.86	0.0316
SEM	11.70	62.40	0.0129

MRM, magnetic resonance microscopy, DV, ductal volume; TV, total volume of the lobe; SD, standard deviation; SEM, standard error of the mean.

* *P *=* *0.0074.

### Statistical analysis

Where noted, standard two-tailed Student’s *t*-tests were performed when comparing values between the wild-type and *cpk* animals. *P *≤* *0.05 were considered significant and indicated by an asterisk (Tables2 and 3).

**Table 3:** Ductal area as determined by histomorphometry

Sample	BDA (mm^2^)	TDA (mm^2^)	BDA/TDA ratio
(A) Wild-type liver (median lobe)			
1	0.023	0.913	0.025
2	0.013	0.802	0.016
3	0.014	0.731	0.019
4	0.019	0.865	0.022
5	0.015	0.559	0.027
6	0.014	0.483	0.029
Mean	0.016	0.726	0.023*
SD	0.004	0.171	0.005
SEM	0.002	0.070	0.002
(B) Mutant liver (median lobe)			
1	0.400	1.526	0.262
2	0.767	1.780	0.431
3	0.751	2.013	0.373
4	0.819	2.002	0.409
5	0.192	0.597	0.322
6	0.965	2.509	0.385
Mean	0.649	1.738	0.364*
SD	0.291	0.646	0.062
SEM	0.119	0.264	0.025

BDA, bile duct area; TDA, total duct area; SD, standard deviation; SEM, standard error of the mean.

* *P *≤* *0.0001.

Bland–Altman analysis of the volume measurements obtained using water displacement versus MRM was performed (Table1, Fig.2B). Concordance (Rho_c), Pearson’s *r* (Pr(r)), and accuracy (C_b) values were all calculated using Stata 13 software (Wang et al. 2008).

## Results

### MRM and volumetric analysis of wild-type and *cpk* mouse kidneys

**Figure 1:**
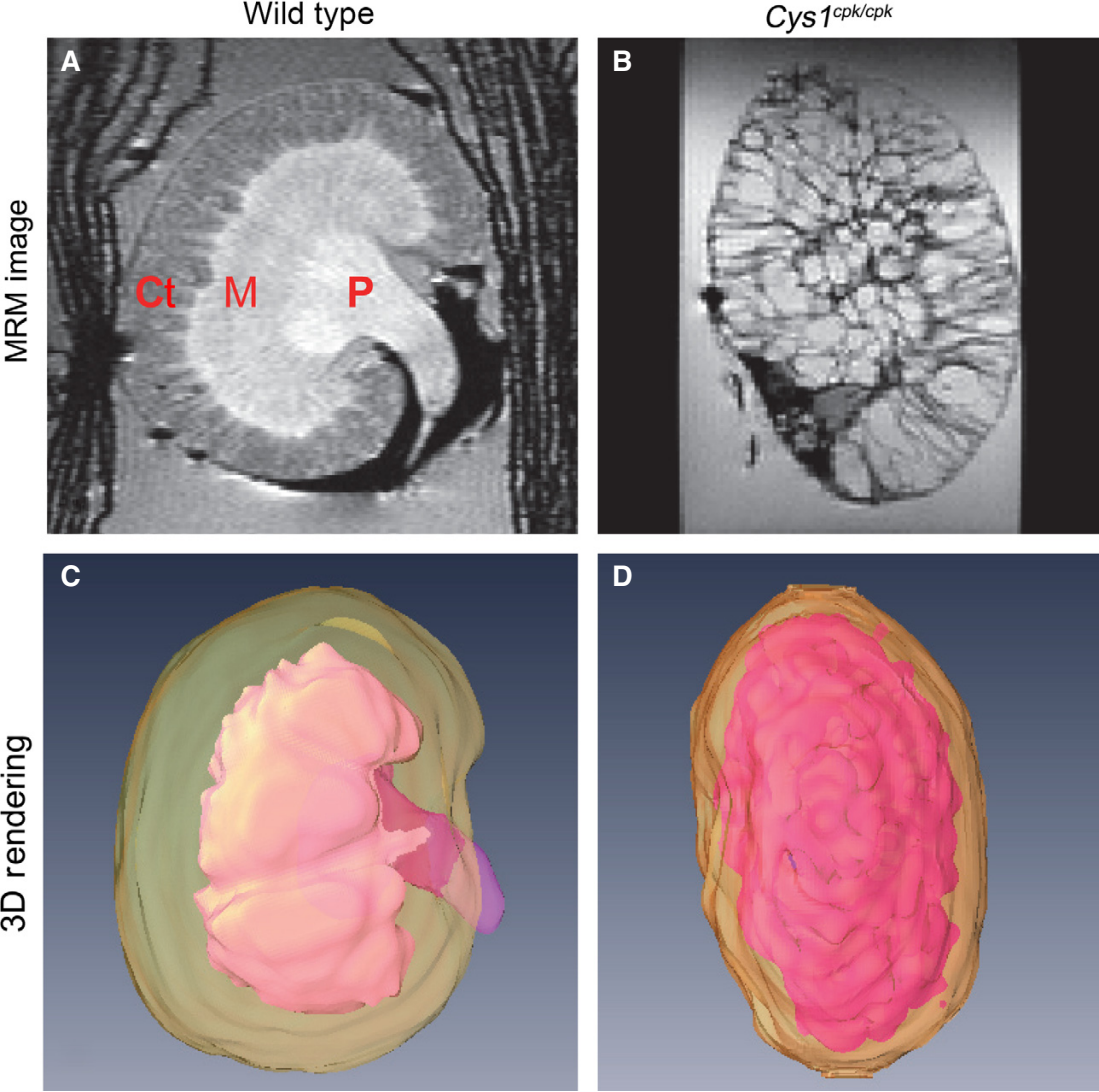
Magnetic resonance microscopy (MRM) and 3D rendering of wild-type and *cpk* kidneys. Representative MRM images of (A) wild-type and (B) *cpk* kidneys. Three-dimensional rendering of the (C) wild-type and (D) *cpk* kidneys are shown. The hyperintense papilla (P) and medulla (M) are shown in pink and the hyopintense cortex (Ct) is in sage green (C) although these delineations are less distinct in the *cpk* sample (D). Images are not shown to scale; (A) and (C), as well as (B) and (D), are corresponding samples. The Kimwipe used to secure the samples appears as dark lines on either side of the kidney in (A).

Kidneys of age-matched *cpk* and wild-type littermates were analyzed by MRM. Representative images from the 2D analysis of wild-type (Fig.1A) and *cpk* (Fig.1B) mice are shown. In Figure1A, the papilla (P) and medulla (M) are visible due to their higher signal intensities (Fig.1C, pink) compared to the cortex (Ct) with a lower signal intensity (Fig.1C, sage green). In Figure1B, the fluid filled cysts characteristic of recessive PKD are visible. Note that the kidneys are so cystic that there are no recognizable anatomic structures/delineations (Fig.1B and D). The compiled 3D renderings are shown in panels C and D.

**Figure 2:**
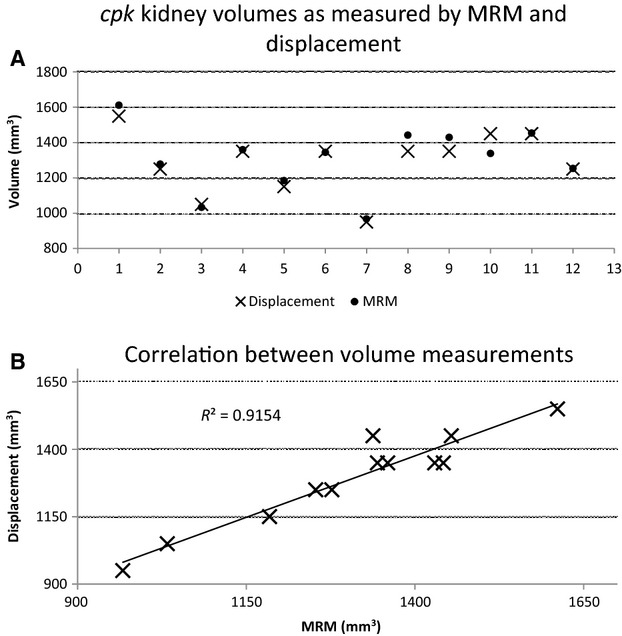
Comparison of magnetic resonance microscopy (MRM) and water displacement for determining the volume of *cpk* kidneys. The volume of the 12 *cpk* kidneys as measured by water displacement (X) and calculated from the MRM analysis (•) are graphed simultaneously in (A). A scatter plot reveals a strong positive correlation between the water displacement and MRM values with an *R*^2^ value of 0.9154 (B).

Renal volume is a common parameter used to assess disease severity. In previous studies from our group renal volume was measured postmortem using water displacement (Mrug et al. 2005). In the current study, the volume was calculated from the 3D renderings (see Materials and Methods section) and compared to water displacement measurements. The average volume for the *cpk* kidneys (*n *=* *12) as determined by MRM was found to be ∼14 times greater than the wild-type (*n *=* *8) (1307 ± 181 mm^3^ vs. 93 ± 10 mm^3^, Table1). As measured by water displacement, the average volume of a *cpk* kidney was 1291 ± 172 mm^3^, which is very similar to the MRM derived value (Fig.2A). Statistical analysis demonstrated excellent concordance (>0.95) between the renal volumes obtained by the MRM data and by water displacement, in terms of actual measurements and reproducibility. The correlation between the two measurement types is shown in Figure2B, the fitted line has a slope of 0.9154. In sum, the volume measurements calculated from our MRM data are as accurate as those determined by water displacement and MRM serves as a precise alternative means of determining volume. In addition, MRM creates a digital record that can be revisited and is noninvasive, potentially allowing for iterative measurements in living animals.

### MRM and 3D rendering of biliary architecture in wild-type and *cpk* mouse livers

**Figure 3:**
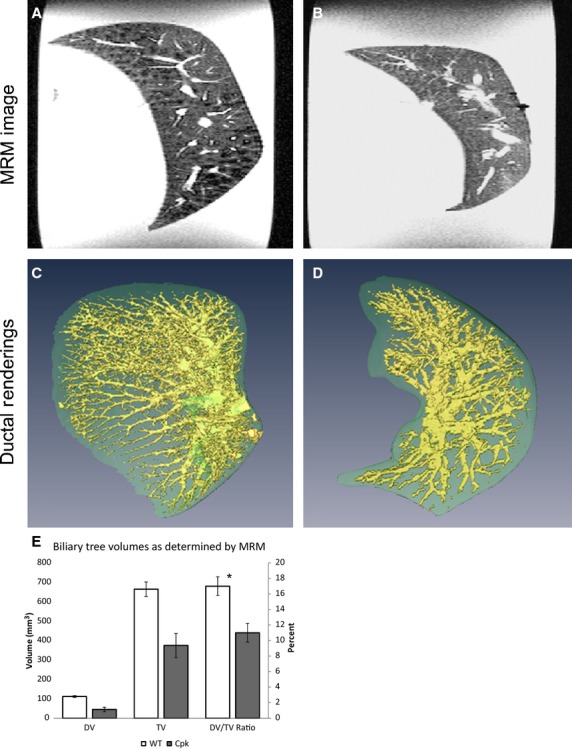
Magnetic resonance microscopy (MRM) and 3D rendering of wild-type and *cpk* livers. Representative MRM images of (A) wild-type and (B) *cpk* livers (median lobe). Three-dimensional rendering of the (C) wild-type and (D) *cpk* livers are shown, where the PBS-infused ducts are shown in yellow. The MRM calculated total volume (TV, of the median lobe), ductal volume (DV), and their ratio are graphically represented in (E). The raw data (as shown in Table2) are represented on the left axis; the right axis shows the ratio (DV/TV) as a percent, asterisk indicates a significant difference between the wild-type and *cpk* ratio as determined by two-tailed Student’s *t*-test, error bars indicate SEM. Images not at the same scale; (A) and (C), as well as (B) and (D), are corresponding samples.

Similar MRM-based analysis of the median liver lobe and its biliary tree were performed. Figure3 shows representative 2D images from both wild-type (Fig.3A) and *cpk* (Fig.3B, not to scale) livers. With 3D rendering, we were able to clearly discern the difference between the PBS-infused bile duct system and the neighboring liver tissue (Fig.3C and D). Significant alterations in the branching structures within the *cpk* liver were distinctly evident. The biliary tree of the *cpk* liver exhibits less extensive and less dense arborization, suggesting that the ductal plate malformation involves a severe developmental defect in ductal branching and the associated vascular elements. Using the same volumetric extraction method we measured the volume of the ductal structure versus the median lobe as a whole (Tables2 and 3 and Fig.3E). The volume of the median lobe of wild-type mice was greater than the age-matched *cpk* samples by almost twofold (wild-type TV: 665 ± 92 mm^3^; *cpk* TV: 374 ± 153 mm^3^; Table2 and Fig.3E). While the *cpk* samples were somewhat variable in size, the largest *cpk* sample was still smaller than the mean volume of the wild type (Table2). The volume of the ducts also reflected this size differential (wild-type DV = 112 ± 11 mm^3^; *cpk* DV = 45 ± 29 mm^3^; Table2 and Figure3E). However, the ratio of ductal volume to total lobe volume was 0.17 ± 0.01 in the wild-type and 0.11 ± 0.01 in the *cpk* livers, indicating that not only was the *cpk* liver reduced in total volume, but also the ductal volume was disproportionately smaller (DV/TV ratio in Table2 and Fig.3E).

### Histopathological analysis of wild-type and *cpk* mouse livers

**Figure 4:**
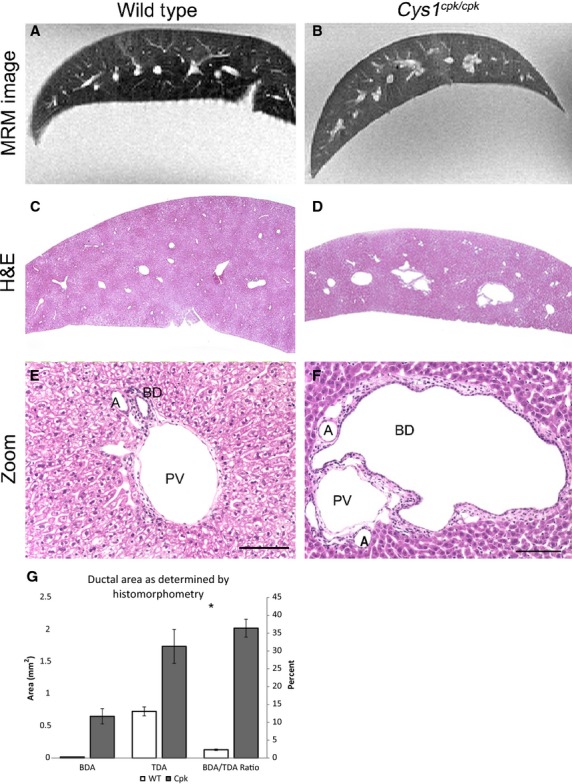
Comparison of magnetic resonance microscopy (MRM) and histopathological analysis of the biliary architecture in wild-type and *cpk* livers. Representative MRM images of (A) wild-type and (B) *cpk* livers are shown with comparative histology after staining with hematoxylin and eosin (H&E, C–F). At higher magnification (×20), the portal vein (PV), arteries (A), and biliary ducts (BD) can be seen. Using histomorphometry, the area of the bile ducts (BDA) as well as the total ductal volume (TDA, the combined areas of the PV, A, and BD) was determined. Graphical analysis of the ductal areas and their ratio are shown in (G). The raw data (as shown in Table3) are represented on the left axis; the right axis shows the ratio (BDA/TDA) as a percent, asterisk indicates a significant difference between the wild-type and *cpk* ratio as determined by two-tailed Student’s *t*-test, error bars indicate SEM. Scale bars in (E) and (F) indicates 100 *μ*m. Panel A is the same sample as in C and E, panel B is the same sample as in D and F; A and B not to scale.

Finally, we compared the quality and resolution of our MRM data to histological data. While the overall organ and ductal dimensions can be seen in the images from MRM, the resolution was not high enough to distinguish specific morphological abnormalities in the epithelial structures versus the vascular elements, versus the ductal plate institium as can be observed with histological analyses (Fig.4A and B vs. E and F). Histomorphometric analyses (Fig.4E and F) revealed that in the wild-type samples, the portal vein and hepatic artery were in close proximity to each other. On the basis of our morphometric analyses, we determined that wild-type bile duct to total ductal area ratio was 0.23 (Table3 and Fig.4G). In comparison, the *cpk* liver had dramatically enlarged bile ducts that expanded to distort the portal vein (Fig.4F). Indeed, the average bile duct area (BDA) in *cpk* livers was ∼40 times larger than in wild-type livers (wild-type BDA: 0.016 ± 0.002 mm^2^; *cpk* BDA: 0.65 ± 0.12 mm^2^; Table3 and Fig.4G), and had a bile duct to total ductal area ratio of 0.364 (Table3 and Fig.4G). The total ductal area in *cpk* liver was also larger than in the wild-type liver (wild-type TDA: 0.73 ± 0.07 mm^2^; *cpk* TDA: 1.74 ± 0.26 mm^2^; Table3 and Fig.4G). Together these data indicate that the primary structure that is enlarged and identifiable in the 3D liver renderings of the *cpk* livers (Fig.3) is the biliary tree.

## Discussion

MRI has been proposed as a noninvasive method for monitoring disease progression in children with PKD (Cadnapaphornchai et al. 2011). MRI acquisition methods are also becoming rapid enough for use in infants, and 3D rendering can be as informative as CT imaging. In experimental models of ADPKD, multiple studies have demonstrated the reliability of MRI studies for monitoring kidney and cyst volume progression (Sun et al. 2002; Reichardt et al. 2009; Zhou et al. 2010). While MRI is used as an imaging modality for some children with ARPKD (Turkbey et al. 2009), the role of MRM in monitoring disease progression, either in human patients or in experimental models, has not been rigorously examined.

Here, we present the first comprehensive study evaluating the reliability of MRM in a recessive PKD mouse model. Our 3D MRM analysis reveals that the average volume of a *cpk* kidney at 3 weeks of age was ∼14 times larger than a wild-type kidney. In addition, we show that 3D volumetric measurements extracted from the MRM data are reliable for determining organ volume.

We found that the MRM analysis was able to readily differentiate between the PBS-infused ductal structures and the neighboring liver tissue in wild-type and *cpk* samples. Thus, for the first time, we were able to generate a 3D rendering of the intact biliary tree in wild-type and *cpk* livers. We note that the *cpk* liver was reduced in size with dilated ductal structures that were less arborized when compared to wild-type livers. Furthermore, while the *cpk* liver was smaller than the wild-type liver, the ductal structures were disproportionately smaller than the total liver size would account for. These data suggest that the ductal plate malformation disrupts the arborization of the entire biliary tree, as well as the terminal differentiation of individual bile ductules (Somlo and Guay-Woodford 2009). Our 3D renderings of MRM images are more robust than 2D morphometric analyses of the full biliary architecture and therefore, we propose that MRM may be a complimentary, noninvasive, and rigorous tool for phenotypic quantitation of the biliary lesion in recessive PKD (Mrug et al. 2005).

We note that the ductal complex in the *cpk* liver were 40-fold larger than those in the wild-type samples, suggesting that the structures represented in the 3D renderings (Fig.3) are most likely bile ductules. These observations and similar studies in human ARPKD livers (Gunay-Aygun et al. 2013) have previously been interpreted as indicating that dilatation of the biliary ductal architecture is a significant contributor to the hepatomegaly observed in ARPKD mice and human patients. However, taken together, our data suggest an alternative hypothesis that the ductal plate malformation results in defects in both the arborization of the biliary tree as well as the terminal differentiation of individual bile ductules. These defects initially cause recessive PKD livers to be smaller in total volume than the wild-type liver. We speculate that the development of hepatomegaly is the consequence of fibrosis associated with the ductal plate malformation, rather than the primary result of the epithelial ductal defect, and develops over time. Indeed, recent analysis of human fetal ARPKD livers indicates that biliary dysgensis is observed in the absence of periportal fibrosis (R. Wells, unpubl. data).

In mice, it is well established that defects in a single gene will manifest variable phenotypes when expressed on different genetic backgrounds. For example, the liver phenotype observed in *cpk* mice on the BALB/c background is virtually absent on the C57BL6/J background (Guay-Woodford et al. 2000a; Guay-Woodford 2003). This observation, coupled with evidence for phenotypic variability within ARPKD sibships (reviewed in (Somlo and Guay-Woodford 2009)), has led to the widely accepted hypothesis that genetic modifiers modulate the phenotypic expression of ARPKD in both human cohorts and experimental models (Woo et al. 1997; Guay-Woodford et al. 2000b; Mrug et al. 2005; Garcia-Gonzalez et al. 2007; O’Meara et al. 2012). Studies from our group have identified *Kif12* as a candidate modifier gene of the renal phenotype in the *cpk* mouse model (Mrug et al. 2005). The same chromosome four interval was also associated with a modulating effect in the severity of the *cpk* liver disease (Mrug et al. 2005). However, the biliary data were not as compelling as the renal data, suggesting the need for more robust tools, such as MRM, to phenotype the liver disease severity.

While the current study was performed in excised tissues from euthanized animals, we now have standardized the protocol for the MRM sequences which we can deploy in sedated living animals in future studies. One potential limitation of this method is that movement and blood flow may impact the clarity of the data in vivo. Indeed, in vivo analysis may require specialized imaging sequences and different contrast methodologies, parameters we plan to optimize in future studies. While CT-based studies such as those used by Masyuk et al. may circumvent this problem (Masyuk et al. 2004), MRM has superior contrast in soft tissue resulting in greater potential for human diagnostic applications. Ultimately, the implementation of technologies such as MRM for serial, iterative structural assessment of the full recessive PKD phenotype in experimental models will provide important insights into disease progression, preclinical treatment efficacy, and the impact of modifier genes in modulating disease progression.

## Conflict of Interest

None declared.
